# Introduction to the special issue: The WHO World Mental Health International College Student (WMH‐ICS) initiative

**DOI:** 10.1002/mpr.1762

**Published:** 2019-01-09

**Authors:** Pim Cuijpers, Randy P. Auerbach, Corina Benjet, Ronny Bruffaerts, David Ebert, Eirini Karyotaki, Ronald C. Kessler

**Affiliations:** ^1^ Department of Clinical, Neuro and Developmental Psychology, Amsterdam Public Health Research Institute Vrije Universiteit Amsterdam Amsterdam The Netherlands; ^2^ Department of Psychiatry Columbia University New York New York; ^3^ Department of Epidemiologic and Psychosocial Research National Institute of Psychiatry, Ramón de la Fuente Muñiz Mexico City Mexico; ^4^ Center for Public Health Psychiatry, Department Neurosciences KU Leuven Leuven Belgium; ^5^ Department of Psychology, Clinical Psychology and Psychotherapy Friedrich‐Alexander University Nuremberg‐Erlangen Erlangen Germany; ^6^ Department of Health Care Policy Harvard Medical School Boston Massachusetts

**Keywords:** affective disorders, depression, effectiveness research, prevention, psychotherapy

## Abstract

Most mental disorders have their first onset in early adulthood. Epidemiological research, as well as research on preventive and early interventions, is therefore very important. This thematic issue focuses on one of the first systematic attempts to develop such services for college students. The WHO World Mental Health International College Student (WMH‐ICS) initiative is based on the largest and continuously growing epidemiological dataset ever collected in college students. Based on these results, the initiative has now started to implement internet‐based interventions for common mental disorders and emotional problems. In this special issue, a general paper about the initiative is presented, as well as a paper on the implementation of the WMH‐ICS initiative in low and middle income countries. It also includes several papers with core epidemiological results of the initiative, a meta‐analysis of internet‐based interventions for mental health problems in college students and the first results of trials conducted as part of the initiative. Taken together, the papers in this special issue show that WMH‐ICS is on its way to becoming a major initiative in addressing the problem of unmet need for treatment of mental health problems among college students.

Most mental disorders have their first onset in early adulthood (De Girolamo, McGorry, & Sartorius, 2019). Epidemiological research and research on preventive and early interventions are therefore very important. New technologies make it possible to conduct epidemiological research relatively easily through online surveys, and a growing number of randomized trials have shown that psychological interventions can be delivered effectively and efficiently through internet‐based and mobile interventions as well (Ebert et al., 2018). Colleges and universities are an excellent setting to conduct epidemiological and intervention research making use of these technologies to prevent or intervene early in mental disorders.

The WHO World Mental Health International College Student (WMH‐ICS) initiative is one of the first systematic attempts to carry out these kinds of research with college students. Starting with a series of mental health needs assessment surveys initiated at KU Leuven in 2012 and growing into a coordinated series of ongoing surveys of this sort across a number of countries that are discussed in this special issue, the initiative has documented the high prevalence (Auerbach et al., 2016, 2018), substantial impairment (Alonso, Mortier et al., 2018), and consistently low receipt of treatment of mental disorders across a growing number of colleges and countries. Based on these results, the initiative has now started to implement internet‐based interventions for common mental disorders and emotional problems (Harrer et al., 2018). We expect that this initiative will grow exponentially in the coming years, with more colleges across the world participating and a growing number of interventions that can be offered to students.

Because the WMH‐ICS initiative has now started off with a growing number of epidemiological and intervention studies, this is the right time to publish a thematic issue with overviews of our most important early results. The papers that are part of this thematic issue provide an overview of what has been accomplished already and maps out our planned steps for the coming years.

In the first paper, Cuijpers et al. (this issue) present an overview of the goals of the WMH‐ICS initiative, with a focus on the three main components: the epidemiological basis, the infrastructure for the development and testing of the internet interventions for mental health problems, and the dissemination of evidence‐based interventions in participating colleges.

The initiative has also considerable potential to be implemented in low and middle income (LAMI) countries, where many college students are the first in their families to attend college. Stress can be especially high among such students but mental health treatment resources are typically quite low. In the second paper, Evans‐Lacko and Thornicroft (2018) discuss the opportunities and challenges of expanding the WMH‐ICS work to such settings. The authors describe the rapid increase in college attendance in LAMIs, especially in middle income countries, and why preventive and early interventions are particularly needed in these settings. Based on extensive experience attempting to improve mental health services in LMICs, Evans‐Lacko and Thornicroft make it clear that challenges will exist in attempting to bring WMH‐ICS to LMICs and that flexibility and long‐term planning will be needed to adapt to the specific settings where the initiative will be implemented.

The next four papers focus on the initial wave of epidemiological surveys carried out in WMH‐ICS, which consist of surveys in 19 colleges across eight countries that yielded information on more than 14,000 students. The first of these papers, by Auerbach et al. (2018), presents an overview of the surveys along with information about the prevalence and basic socio‐demographic distributions of common mental disorders in the surveys. Auerbach and colleagues not only show that mental disorders are widely distributed across the student population but also that they commonly co‐occur and that this co‐occurrence may have profound implications for treatment.

The next paper, by Alonso, Vilagut, et al. (2018), documents that mental disorders are strongly associated with the role impairment experienced by students. Indeed, the data reported by Alonso et al. suggest that the majority of role impairments found among college students can be traced back to the mental disorders assessed in our surveys. The societal costs associated with failing to intervene either to prevent or to treat these disorders in a timely fashion are laid out clearly in this paper.

In the next paper, Bruffaerts et al. (this issue) investigate the receipt of treatment among college students with mental disorders. As detailed in that paper, treatment rates are consistently low across all the colleges surveyed in WMH‐ICS. This is true despite the fact that the vast majority of the colleges included in these first WMH‐ICS surveys have student mental health clinics where treatment is available either at low or no cost.

The reasons for this treatment gap are explored in the next paper. Ebert et al. (this issue) examine barriers to treatment reported in the surveys by students. The analysis makes it clear that psychological barriers, such as a preference to handle problems alone, are more important than practical barriers. The authors suggest that internet interventions might help resolve these barriers by providing students with a private way of obtaining treatment.

The next paper, also by Ebert and colleagues (this issue), uses an experimental design to evaluate the potential for customized feedback to increase the willingness of students who screen positive for clinically significant emotional problems to seek treatment. As detailed in the paper, promising results were found suggesting that willingness of students to seek treatment for emotional problems may indeed be increased with simple procedures such as customized feedback addressing psychological barriers to treatment. It is also noted, though, that a wide range of other options exist for improving recruitment by targeting motivational messages to the particular conditions and barriers reported by the students. Based on these results, in conjunction with the finding of high unmet need for treatment among college students in the earlier Bruffaerts et al. paper, we anticipate a long‐term program of experiments along these lines to be carried out in conjunction with the annual WMH‐ICS surveys.

The final two papers focus on e‐health interventions. The first of these papers, by Harrer et al. (this issue), is a meta‐analytic review of randomized trials involving the use of internet‐based interventions for mental health problems among college students. This study shows that a considerable number of such trials have already been conducted and that the results are very encouraging regarding the potential of such interventions to treat such diverse student mental health problems as depression, anxiety, stress, eating disorder symptoms, and sleep problems.

The second intervention paper, by Kählke et al. (2018), presents the results of one of the first randomized treatment trials conducted within the context of WMH‐ICS. That trial focused on social anxiety disorder, a commonly occurring and sometimes seriously impairing disorder among college students. Social anxiety disorder poses special challenges for intervention due to the fact that the symptoms of the disorder create a psychological barrier to treatment that results in only a small minority of the students who suffer from this disorder seeking treatment. The Kählke et al. intervention is unique in that it used mass email advertisements sent to students to recruit extremely shy students who otherwise would not seek treatment to receive confidential help via the internet. The advertisements informed potential subjects that they could receive confidential help via the internet that would not require them to make in‐person treatment visits or, indeed, to speak to a clinician after the initial telephone intake interview. The success of this intervention illustrates the potential value of WMH‐ICS in using innovative outreach and intervention delivery methods to target important pockets of unmet need for treatment among college students with diverse emotional problems.

Taken together, the papers in this special issue show that WMH‐ICS is on its way to becoming a major initiative in addressing the problem of unmet need for treatment of mental health problems among college students. The initiative has a unique combination of features that bode well for its success: ongoing needs assessment surveys, which will allow us to pinpoint areas of unmet need for treatment, monitor barriers to treatment, carry out experiments with diverse intervention recruitment strategies, implement wide‐ranging internet‐based interventions (and possibly subsequent in‐person interventions) that will be evaluated using an effectiveness trial approach, and use evidence‐based dissemination activities that will guard against intervention degradation with ongoing monitoring of both processes and outcomes. Challenges will exist, of course, in growing the initiative, but the focal population is of enormous public health importance, the level of current unmet need for treatment is high, and opportunities for intervention delivery are vast. Based on this unique combination of characteristics, we have every expectation that the initiative will provide to be valuable.

## FUNDING INFORMATION

The Dutch part of this project is financially supported by ZonMw, The Netherlands Organisation for Health Research and Development (grant 636110005).
